# Enhancing Membrane Repair Using Recombinant MG53/TRIM72 (rhMG53) Reduces Neurotoxicity in Alzheimer’s Disease Models

**DOI:** 10.3390/biom15030418

**Published:** 2025-03-15

**Authors:** Hannah R. Bulgart, Miguel A. Lopez Perez, Noah Weisleder

**Affiliations:** 1 Department of Molecular and Cellular Biochemistry, University of Kentucky, Lexington, KY 40536, USA; hannah.bulgart@uky.edu; 2 Department of Physiology and Cell Biology, The Ohio State University, Columbus, OH 43210, USA; lopezperez.3@osu.edu

**Keywords:** Alzheimer’s disease, plasma membrane repair, rhMG53, protein therapeutics, neurotoxicity

## Abstract

Alzheimer’s Disease (AD) is the most common neurodegenerative disease that involves neuronal cell death initiated by the breakdown of the plasma membrane. Amyloid beta (Aβ), a hallmark protein that contributes to AD pathogenesis, is known to interact directly with the plasma membrane and induce increased intracellular calcium levels, reactive oxygen species (ROS), and cell death. Our recent studies revealed that elevated levels of Aβ_42_ induce a plasma membrane repair defect in neurons that compromises this conserved cellular response that would normally repair the disruption. Here, we tested if recombinant MG53/TRIM72 protein (rhMG53), a therapeutic protein known to increase plasma membrane repair capacity, could enhance membrane repair in AD neurons. rhMG53 increased plasma membrane repair in ex vivo and in vitro tissue treated with Aβ_42_ or cerebrospinal fluid from AD patients, normalizing intracellular calcium levels, ROS, and cell death in treated cells. This study demonstrates that increasing plasma membrane repair can rescue neural cells from the neurotoxic effects of Aβ, indicating that elevating plasma membrane repair could be a viable therapeutic approach to reduce neuronal death in AD.

## 1. Introduction

Alzheimer’s Disease (AD) is a progressive neurodegenerative disease leading to the death of neuronal cell types in the hippocampus and neocortex of the brain, reduced memory, and the death of the patient [1,2,3,4]. There are limited treatment options available for AD, with only monoclonal antibody treatments approved in the last two decades. One hallmark protein accumulation in AD is the increased production of amyloid beta (Aβ), which is associated with neurotoxicity and neuronal death. Previous studies have demonstrated a relationship between Aβ and the plasma membrane by which the protein modulates the integrity of the plasma membrane leading to toxic intracellular conditions [5,6,7,8,9]. A recent study from our laboratory demonstrated a membrane repair defect when primary neurons were treated individually with recombinant Aβ_42_ or AD patient cerebrospinal fluid (CSF) with elevated concentrations of Aβ_42_. Furthermore, we utilized a transgenic AD mouse model, APP/PS1, which overexpresses Aβ and recapitulates some of the memory defects observed in AD patients [10]. In ex vivo brain sections from APP/PS1 mice, we observed a significant decrease in membrane repair capacity compared to the control C57Bl/6 brain sections. The observed repair defect was due to a decrease in dysferlin protein expression, a membrane repair protein, and this decrease in dysferlin contributes to neurotoxicity and cell death [11]. We hypothesize that cell death in AD results from a membrane repair defect, at least in part, and therapeutically enhancing membrane repair capacity in AD neurons could reduce neurotoxicity.

Plasma membrane repair is an essential cellular function to restore the barrier function of the membrane, normalize ionic gradients, and allow the cell to survive the injury [12,13]. There are several mechanisms involved in membrane repair, but the most commonly studied mechanism is called patch formation. Patch formation is initiated by extracellular calcium flowing through a membrane injury site which initiates intracellular vesicles to traffic to the injury site where they can fuse with other vesicles and the plasma membrane to patch the disruption. One of the proteins that is embedded in those intracellular vesicles is mitsugumin 53/tripartite motif protein 72 (MG53/TRIM72). MG53/TRIM72 is highly expressed in striated muscle, but not at high levels in the nervous system [14,15,16]. In addition to membrane repair, TRIM72 functions in calcium signaling [17], vesicle trafficking [15], and anti-inflammatory pathways [18]. Expressing MG53/TRIM72 in cell types where it is not natively expressed, including cells from the nervous system, recapitulates its function in increasing membrane repair capacity [19]. Recombinant human MG53/TRIM72 protein (rhMG53) has been used as a therapeutic approach to increase membrane repair and improve pathology in striated muscle where MG53 is natively expressed [19,20,21] and also in tissues where MG53/TRIM72 is not natively expressed, including neurons [22,23,24]. Previous studies have shown that rhMG53 translocates to injury sites and binds to phosphatidylserine to increase membrane repair capacity, which restores membrane integrity and minimizes cell death [16,19]. Previous studies have shown that rhMG53 can be an efficacious therapeutic approach in models of muscular dystrophies [19,20], cardiac ischemia [21], and lung fibrosis [25]. Our studies also show that rhMG53 can increase membrane repair in cells where dysferlin levels are decreased, including muscle from dysferlin knockout mice [20]. In the present study, we tested the therapeutic efficacy of rhMG53 in models of AD and observed enhanced neuronal membrane repair, reduced neurotoxicity, and limited cell death in ex vivo APP/PS1 mouse brain tissue and Aβ_42_-treated cultured neuronal cell types.

## 2. Materials and Methods

### 2.1. Cell Culture and Recombinant Proteins

Mouse neuroblastoma cells (N2A) (ATCC #CCL-131) were maintained at 37 °C, 5% CO_2_, and cultured in DMEM (Thermo Fisher Scientific, Waltham, MA, USA) supplemented with 10% FBS (VWR, Radnor, PA, USA), 1× penicillin-streptomycin (Gibco, Waltham, MA, USA #15140122), and GlutaMAX (Gibco, Waltham, MA, USA). Primary Sprague Dawley rat cortex neurons (Thermo Fisher Scientific, Waltham, USA) were plated on 0.01% poly-L-ornithine (Sigma-Aldrich, St. Louis, MO, USA)-coated plates, maintained at 37 °C, 5% CO_2_, and cultured in Neurobasal Plus media (Thermo Fisher Scientific, Waltham, MA, USA), 2% B-27 Plus supplement (Thermo #A3582801), and 0.5 mM GlutaMAX (Gibco, Waltham, MA, USA). The cell cultures were treated with 1 µM lyophilized human monomeric amyloid β-peptide (1–42) (R&D Systems, Minneapolis, MN, USA) overnight and/or recombinant human MG53/TRIM72 produced in the laboratory using *E. coli* [19] that was reconstituted in phosphate-buffered saline (137 mM NaCl, 2.7 mM KCl, 10 mM Na_2_HPO_4_, 1.8 mM KH_2_PO_4_).

### 2.2. Patient Cerebrospinal Fluid Samples

Alzheimer’s Disease cerebrospinal fluid (CSF) samples were obtained and deidentified from The Ohio State University Neuroscience Research Institute Brain Bank and Biorepository under protocols approved by The Ohio State University Institutional Review Board (IRB #2022H0127).

### 2.3. Mice

APP/PS1 (#034829-JAX) and C57Bl/6 (#000664) mice were purchased from The Jackson Laboratory and housed under standard conditions. All procedures were approved by The Ohio State University Institutional Animal Care and Use Committee (IACUC).

### 2.4. Infrared Laser Damage Assay

Cell cultures were grown on 35 mm glass bottom plates (MatTek, Ashland, MA, USA), at a concentration of 100,000 cells per milliliter, prior to experimentation. Just prior to experiments, cell culture media was aspirated, washed twice with 1× Tyrdoes buffer (137 mM NaCl, 2.7 mM KCl, 2 mM CaCl_2_, 0.2 mM Na_2_HPO_4-_7H_2_O, 1 mM MgCl_2_, 12 mM NaHCO_3_), and supplemented with fresh buffer and 10.5 µM FM4-64 (Thermo #T13320). Experiments involving patient CSF samples at 1:100 dilution and recombinant MG53 protein were added directly after fresh buffer supplementation and FM4-64 supplementation. For experiments involving brain tissue, the brain tissue was adhered to the previously mentioned glass bottom plates with liquid bandage, and 1 mL Tyrodes buffer and 10.5 µM FM4-64 were added directly to the brain tissue. A FluoView FV1000 MPE (Olympus, Shinjuku City, Tokyo, Japan) confocal laser microscope was used to conduct the assay. A portion of the plasma membrane was selected and damaged at 40–60% maximal laser power. Twenty total frames, every 3 s, were taken for each injury—3 frames prior to injury and 17 frames following the injury to track repair capacities. The fluorescence of the injury site was measured as follows: mean fluorescence of injury—mean fluorescence of the background/mean fluorescence of background notated as ΔF/F0 per timepoint.

### 2.5. Neurotoxicity Assays

Just prior to experimentation, cell culture media was removed, washed twice with pre-warmed 1× Tyrodes buffer (137 mM NaCl, 2.7 mM KCl, 2 mM CaCl_2_, 0.2 mM Na_2_HPO_4-_7H_2_O, 1 mM MgCl_2_, 12 mM NaHCO_3_), and replaced with fresh pre-warmed 1× Tyrodes buffer. Cell death: A total of 500 nM propidium iodide solution was added to each well and imaged with EVOS FL Auto 2 microscope brightfield and Texas Red channels. Propidium iodide positive cell percentage was calculated by dividing the propidium iodide positive cells by the total cell count per biological replicate. Intracellular calcium: Intracellular calcium levels were determined using Fluo-4 Calcium Imaging Kit (Thermo #F10489). Then, 2 mL of the 1× Fluo-4 AM and PowerLoad solution was added to each well and incubated at 37 °C for 30 min followed by 30 min at room temperature. The staining solution was removed and the cells were washed once with 1× Tyrodes buffer and supplemented with fresh 1× Tyrodes buffer for imaging. The cell cultures were imaged with the GFP channel on EVOS FL Auto 2 microscope (Thermo Fisher Scientific, Waltham, MA, USA). Cytosolic calcium levels were calculated by measuring the intracellular mean fluorescence intensity subtracted by the average background mean fluorescence intensity and divided by the average background mean fluorescence intensity (ΔF/F0). Oxidative stress: Cytosolic oxidative stress detection was completed with the CellROX Deep Red probe (Thermo #C10422). Then, 5 µM CellROX was added directly into the cell culture media and incubated for 30 min at 37 °C. The cell culture media was aspirated and the cells were washed thrice with 1× Tyrodes buffer and supplemented with fresh 1× Tyrodes buffer for imaging. The cells were imaged with EVOS FL Auto 2 microscope on the Texas Red channel. Oxidative stress levels were determined by measuring the intracellular mean fluorescence intensity subtracted by the average background mean fluorescence intensity and divided by the average background mean fluorescence intensity (ΔF/F0).

### 2.6. Statistical Analyses

GraphPad Prism (Version 10.1.1, GraphPad, Boston, MA, USA) was used to generate graphs/figures and conduct statistical tests. Unpaired t-tests and one-way ANOVA with Tukey’s multiple comparisons test were used to statistically analyze the datasets. *p* values less than 0.05 were considered significant.

## 3. Results

### 3.1. APP/PS1 Brain Slices Treated with rhMG53 Rescues Repair Capacity

**Figure 1:**
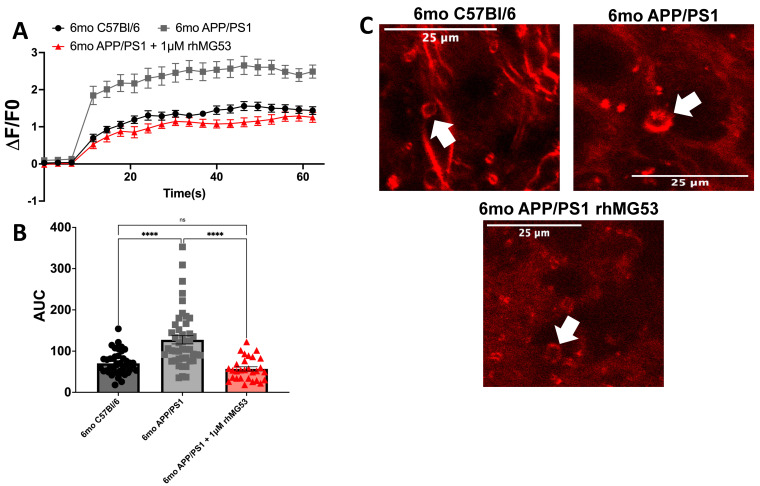
Membrane repair capacity of *ex vivo* APP/PS1 brains is significantly increased with recombinant MG53 treatment. *Ex vivo* 6-month C57Bl/6 and APP/PS1 whole brain slices were treated without or with 1 µM rhMG53. (**A**) Membrane repair kinetics curves, (**B**) Area Under the Curve (AUC) for each injury from (**A**), and (**C**) representative injuries with arrows indicating injury sites (n = 29–45 injured cells, ANOVA, F_(2113)_ = 7.981, **** *p* < 0.0001; Tukey’s: 6mo C57Bl/6 vs. 6mo APP/PS1, **** *p* < 0.0001; 6mo C57Bl/6 vs. 6mo APP/PS1 + 1 μM rhMG53, *p* = 0.4765; 6mo APP/PS1 vs. 6mo APP/PS1 + 1 μM rhMG53, **** *p* < 0.0001).

Our previous published work demonstrated an induced plasma membrane repair defect with the treatment of recombinant Aβ_42_ and AD patient CSF to primary neurons and ex vivo APP/PS1 brain tissue [11]. To test the efficacy of rhMG53 on enhancing plasma membrane repair in APP/PS1 mice, an AD mouse model which overexpress Aβ, we treated live whole brain slices from 6-month APP/PS1 mice, with no experimental intervention or 1 µM rhMG53 and compared the data to 6-month C57Bl/6 control mouse whole brain slices. We chose to use 1 µM rhMG53 because it was previously shown to be a highly effective dose to increase membrane repair, but not induce toxicity [19,23]. We tested the relative membrane repair capacities of neurons identified during confocal microscopy of these treated brain slices with an established laser membrane damage assay where cell membranes were ablated with a multi-photon infrared laser in the presence of FM4-64, which binds to the membrane at the damage sites, and dye influx ceases when the membrane reseals [26]. We observed a significant decrease in membrane repair capacity, marked by an increase in FM4-64 dye fluorescence at injury sites in 6-month APP/PS1 brain slices with no experimental intervention compared to 6-month C57Bl/6 brain slices, as we have previously demonstrated [11]. Treatment of 1 μM rhMG53 to 6-month APP/PS1 brain slices significantly increased membrane repair capacity compared to 6-month APP/PS1 brain slices with no treatment, and no significant differences compared to the 6-month C57Bl/6 brain slices, indicating a return to baseline repair kinetics (Figure 1). Here, we conclude that rhMG53 can therapeutically increase membrane repair capacity in ex vivo APP/PS1 brain tissue.

### 3.2. Exogenous rhMG53 Enhances Membrane Repair in Aβ_42_ and Patient CSF-Treated Neurons

**Figure 2:**
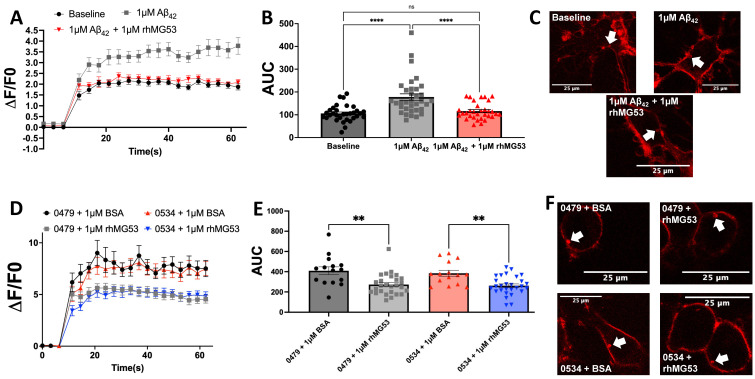
rhMG53 treatment increases membrane repair capacity in Aβ_42_ and AD patient CSF treated neural cells. (**A**–**C**) Primary neurons were pre-treated for 16 h prior to experimentation with 1 µM Aβ_42_ and/or 1 µM rhMG53. (**A**) Membrane repair kinetics, (**B**) Area Under the Curve (AUC) from each injury from (**A**), and (**C**) representative injuries with arrows indicating the injury sites (n = 32–34 injured neurons; ANOVA, F_(2,95)_ = 4.977, **** *p* < 0.0001; Tukey’s: baseline vs. Aβ_42_, **** *p* < 0.0001; baseline vs. Aβ_42_ + 1 µM rhMG53, *p* = 0.7518; Aβ_42_ vs. Aβ_42_ + 1 µM rhMG53, **** *p* = 0.0001). (**D**–**F**) Mouse neuroblastoma cells were pre-treated for 16 h prior to experimentation with 1:100 dilution of AD CSF plus 1 µM BSA or 1 µM rhMG53. (**D**) Membrane repair kinetics, (**E**) Area Under the Curve (AUC) from each injury from (**D**), and (**F**) representative injuries with arrows indicating injury sites (n = 15–28 injured cells; ANOVA, F_(5131)_ = 0.6705; *p* < 0.0001; Tukey’s: 0479 + BSA vs. 0479 + rhMG53, ** *p* = 0.0021; 0534 + BSA vs. 0534 + rhMG53, ** *p* = 0.0075).

To directly test the efficacy of rhMG53 on enhancing plasma membrane repair in primary neurons, we challenged primary rat cortex neurons with 1 µM Aβ_42_ exposure to simulate the effects of AD and then measured membrane repair by laser membrane damage assay. As with our previous published studies [11], we observed a significant increase in dye fluorescence at the injury sites in Aβ_42_-treated neurons compared to the baseline controls. With the addition of rhMG53 to Aβ_42_-treated neurons, we observed a significant decrease in dye fluorescence at the injury sites compared to Aβ_42_ treatment alone, and no significant differences between the saline control, indicating a return to baseline membrane repair kinetics (Figure 2A–C). Additional studies tested the efficacy of improving membrane repair when mouse neuroblastoma cells (N2A) were exposed to AD patient CSF with the application of 1 µM bovine serum albumin (BSA) as a control or 1 µM rhMG53. Under these conditions, the high concentrations of native Aβ_42_ in AD patient CSF can compromise membrane repair in otherwise healthy neurons [11]. We exposed N2As with a 1:100 dilution of AD patient CSF with high concentrations of Aβ_42_ (>1000 pg/mL) plus 1 µM BSA or 1 µM rhMG53 and tested the treated cells with the laser membrane damage assay. The addition of rhMG53 to AD CSF-treated cells significantly increased plasma membrane repair capacity compared to cells treated with patient CSF and BSA (Figure 2D–F). Together, these data demonstrate the ability for rhMG53 to significantly increase membrane repair capacity in neuronal cell cultures treated with recombinant Aβ_42_ or AD patient CSF samples.

### 3.3. rhMG53 Reduces Neurotoxicity in Aβ_42_-Treated Neuroblastomas

**Figure 3:**
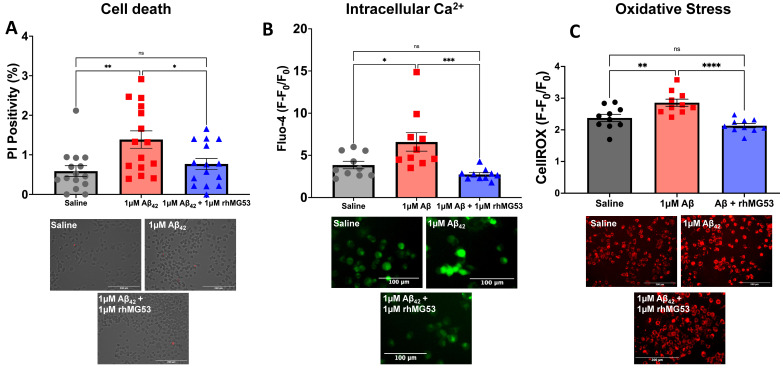
Recombinant MG53 reduces neurotoxicity in Aβ_42_-treated neuroblastomas. N2A cells were pre-treated for 16 h with saline, 1 μM Aβ_42_, or 1 μM Aβ_42_ + 1 μM rhMG53. (**A**) Cell death percentage determined by propidium iodide live cell staining and representative images (n = 15 fields, ANOVA, F_(2,42)_ = 3.778, ** *p* = 0.0048, Tukey’s: saline vs. 1 μM Aβ_42_, ** *p* = 0.0051; saline vs. 1 μM Aβ_42_ + 1 μM rhMG53, *p* = 0.7377; 1 μM Aβ_42_ vs. 1 μM Aβ_42_ + 1 μM rhMG53, * *p* = 0.0352). (**B**) Intracellular calcium levels determined by Fluo-4 calcium imaging kit fluorescence and representative images (n = 10 fields, ANOVA, F_(3,36)_ = 3.468, **** *p* < 0.0001, Tukey’s: saline vs. 1 μM Aβ_42_, * *p* = 0.0144; saline vs. 1 μM Aβ_42_ + 1 μM rhMG53, *p* = 0.5763; 1 μM Aβ_42_ vs. 1 μM Aβ_42_ + 1 μM rhMG53, *** *p* = 0.0004). (**C**) Oxidative stress levels determined by CellROX fluorescence and representative images (n = 10 fields, ANOVA, F_(2,27)_ = 1.131, **** *p* < 0.0001, Tukey’s: saline vs. 1 μM Aβ_42_, ** *p* = 0.0058; saline vs. 1 μM Aβ_42_ + 1 μM rhMG53, *p* = 0.2129; 1 μM Aβ_42_ vs. 1 μM Aβ_42_ + 1 μM rhMG53, **** *p* < 0.0001).

While we find that rhMG53 can enhance membrane repair capacity in both in vitro and ex vivo AD models, we wanted to determine if increasing membrane repair could have therapeutic effects on downstream neurotoxicity markers. To assess neurotoxicity, we treated N2As with saline, 1 µM Aβ_42_, or 1 µM Aβ_42_ plus 1 µM rhMG53 and assessed cell death, intracellular calcium, and oxidative stress levels as representative neurotoxicity markers. With all three neurotoxicity markers, we observed a significant increase in the individual markers with Aβ_42_ treatment compared to the saline control; however, the addition of 1 µM rhMG53 to Aβ_42_-treated cells significantly reduced the neurotoxicity markers to the extent that there was no significant difference between rhMG53-treated cells and the saline control cells (Figure 3). Together, the data presented here demonstrate that increasing membrane repair using rhMG53 can decrease downstream neurotoxicity due to enhancing the integrity of the plasma membrane.

## 4. Discussion

Our studies demonstrate a novel therapeutic approach to reducing neuronal cell death and neurotoxicity in AD neurons. The defect in plasma membrane repair resulting from Aβ_42_ exposure [11] has several downstream consequences. Loss of plasma membrane integrity leads to calcium dyshomeostasis [7], contributing to calcium overload, oxidative stress [8,9], and neuronal death [6]. We hypothesized that increasing membrane repair capacity with rhMG53 would reduce neurotoxicity in AD neurons by enhancing plasma membrane repair and reinforcing the integrity of the membrane. We found that rhMG53 could enhance plasma membrane repair in ex vivo brain slices from APP/PS1 mice (Figure 1), primary rat neurons treated with recombinant Aβ_42_, and N2A cells treated with AD patient CSF (Figure 2). This enhanced plasma membrane repair can decrease downstream neurotoxicity induced by Aβ_42_ exposure (Figure 3). This demonstrates that rhMG53 can therapeutically enhance plasma membrane repair in AD neurons, both in vitro and ex vivo, illustrating the therapeutic potential of agents that can enhance plasma membrane repair and rescue neurotoxicity conditions, as we determined this approach could block the progressive cell death observed in AD. While these studies did show that increasing membrane repair in general, and specifically with rhMG53, could reduce pathologic hallmarks of AD in brain slices, primary neurons, and cultured neuroblastoma cells, we only made use of one AD mouse model and two cultured cell types. This limitation leads us to plan future studies that will utilize additional mouse models, such as the 5XFAD mouse, and other translational in vitro models, like neurons derived from AD patient iPSCs. We hypothesize that increasing membrane repair would prevent necrotic cell death as well as reduce the influx on calcium into neurons and abrogate the cellular damage that would result.

While this is the first study to utilize rhMG53 as a therapeutic approach to increase membrane repair in AD neurons, previous studies by our group and others have used the protein to target neuroinflammation [27] and nerve injury [23,27] in the central and peripheral nervous systems. This reduced neuroinflammation could result from the increased membrane repair and subsequent prevention of cell death, as we show here, which would reduce inflammatory signals released from injured and dying cells. In any case, rhMG53 could have therapeutic potential in treating AD, especially considering reports showing that rhMG53 can pass through the blood–brain barrier [28], supporting that this approach could be effective in AD patients when applied systemically. While there is potential for this approach, there are also some challenges to the use of rhMG53 as a therapy including that the protein was linked to development of metabolic disorders [29,30]. While the extent that MG53 can contribute to metabolic disorders remains unclear [31,32,33], these potential off target effects of rhMG53 are a limitation of this study. However, our studies here clearly demonstrate that there is potential therapeutic value in increasing membrane repair capacity in AD neurons. Fortunately, there are other approaches known to increase membrane repair capacity, with the most relevant of those in this case being to increase the expression of either full length dysferlin [34,35,36] or optimized shorter forms of dysferlin [36,37], an approach that is under clinical development for treatment of dysferlin deficiency in skeletal muscle (NCT05906251). We previously showed that increased dysferlin expression in neurons can restore membrane repair in AD neurons [11]; therefore, these gene therapy approaches may have relevance in the treatment of AD. Other therapeutic approaches could include recombinant protein supplementation of other relevant membrane repair proteins, such as dysferlin or Annexin A6, or gene therapy approaches using the annexin family of genes or TRIM72. In any case, future studies need to address the extent that increasing membrane repair in AD mouse models can prevent the behavioral and memory changes associated with the progression of the disease.

## 5. Conclusions

Our research presented here is the first study to utilize the plasma membrane repair therapeutic, rhMG53, to enhance membrane repair and reduce cell death and neurotoxicity in AD models. Our findings show that rhMG53 can overcome the neuronal plasma membrane repair defect induced by Aβ_42_ treatment in both in vitro and ex vivo models. Importantly, we demonstrated that rhMG53 is a clinically relevant therapeutic approach as rhMG53 was able to significantly increase plasma membrane repair in cell cultures treated with AD patient CSF, which contained high concentrations of Aβ_42_. Overall, these results provide evidence for the therapeutic potential of increasing plasma membrane repair as an approach to slow AD progression. In this respect, this initial study utilizing rhMG53 as a therapy to combat the toxic effects of Aβ_42_ in AD neurons points towards future therapies for AD.

## Data Availability

The datasets used and/or analyzed during the current study are available from the corresponding author on reasonable request.

## Abbreviations

The following abbreviations are used in this manuscript:

Aβ	Amyloid beta
AD	Alzheimer’s Disease
AUC	Area Under the Curve
BSA	Bovine serum albumin
CSF	Cerebrospinal fluid
N2A	Mouse neuroblastoma
rhMG53	Recombinant human MG53
